# Unguided temporary pacing via jugular/subclavian vein in an emergency department of a high-volume tertiary care hospital of India: its safety, efficacy, and practicability

**DOI:** 10.1186/s43044-022-00271-z

**Published:** 2022-04-25

**Authors:** Najeeb Ullah Sofi, Santosh Kumar Sinha, Araf Ali, Siddharth Samrat, Mahmodullah M. Razi, Awadhesh Kumar Sharma, Mohit Sachan, Umeshwar Pandey, Ramesh Thakur

**Affiliations:** 1 Department of Cardiology, LPS Institute of Cardiology, GSVM, GT Road, Swaroop Nagar, Kanpur, UP 208002 India; 2 Department of Ophthalmology, SKIMS Medical College, Srinagar, Kashmir India; 3 Department of Cardiology, Post Graduate Institute of Child Health, Noida, India

**Keywords:** Temporary pacing, Heart block, Bradycardia, Myocardial infarction, Internal jugular vein

## Abstract

**Background:**

Temporary pacing is usually performed by cardiologists under fluoroscopic, echocardiographic, or ECG guidance. However, in the developing world, there are inadequate number of cardiologists, and C-arm, catheterization laboratories, or echocardiography are not available at primary or secondary healthcare facilities. In addition, in emergencies option of fluoroscopy and echocardiography is limited. So these patients are transferred to a facility where cardiologists and these facilities are available. Crucial time is lost in transit, which leads to increased mortality. In this study, we aimed to evaluate the safety, efficacy, and practicability of unguided temporary pacemaker insertion.

**Results:**

A total of 1093 patients were enrolled in this study. After cannulating the internal jugular vein or subclavian vein, the pacing lead attached to the pulse generator was advanced blindly till ventricular pacing was achieved. Procedural success was taken as the primary endpoint. Secondary endpoints included the number of attempts taken for successful central venous puncture and procedural time. Complications and mortality were assessed for safety outcomes. Finally, the position of the pacing lead was assessed after the procedure on X-ray or fluoroscopy. The procedure was successful in all but one patient in whom a femoral vein approach was required because of brachiocephalic vein obstruction. Right internal jugular access was achieved in 981 (89.75%) patients. The mean number of attempts taken for achieving successful venous accesses was 1.54 ± 0.85; however, in 726 (66.42%) patients it was achieved in the first attempt. The mean procedural time was 11.5 ± 2.1 min. Overall, 117 (10.70%) patients developed complications; however, most of them were minor. Pneumothorax developed in 12 (1.1%) patients, of whom 2 needed an intercostal tube. Pericardial effusion was seen in 21 (1.92%) patients. Pacing lead tip was located in the right ventricular cavity abutting interventricular septum or free wall in 843 (77.20%) patients. No mortality attributable to procedure occurred.

**Conclusions:**

Unguided temporary pacing via jugular or subclavian venous approach in an emergency setting is possible with high success and a low complication rate. Thus, it is a safe and effective procedure, and clinicians working at primary and secondary healthcare levels should be encouraged to perform this procedure.

*Trial registration* UMIN Clinical Trials Registry, UMIN000046771. Registered 28 January 2022—Retrospectively registered, https://center6.umin.ac.jp/cgi-open-bin/ctr_e/ctr_view.cgi?recptno=R000053348

## Background

Temporary pacing is needed in symptomatic bradycardias of various etiologies either as a bridge to permanent pacing or till recovery of spontaneous rhythm. It is done via a jugular, subclavian, or femoral venous approach either under fluoroscopic, echocardiographic, or under ECG guidance [1, 2]. However, in most of the developing world, this procedure is done blindly. Limited resources, expertise, and time constraints are the most important factors which restrict the use of the imaging while performing any procedure. In the developing world, there are not an adequate number of cardiologists, and also C-arm, catheterization laboratories, or echocardiography are not available at primary or secondary healthcare facilities. So the patients are transferred from these centers to a facility where cardiologists and these facilities are available. Crucial time is lost in transit, which leads to increased mortality.

## Methods

### Study design and patient population

In this open-label, single-arm, single-center study, a total of 1093 patients, who presented to our emergency room and had any indication for urgent pacing, were enrolled between February 2020 and August 2021. Procedures were done by the second and third-year cardiology fellows in the emergency room.

The protocol of the study was approved by the institutional Ethics Committee. Pre-procedural written and informed consents were obtained from all the patients or their legally authorized guardians.

### Procedural details

ECG electrodes were attached to the patient’s chest and were connected to the monitor. The patient’s head was turned opposite to the side from which access was planned. The neck was cleaned and draped. The internal jugular vein (IJV) was cannulated with a 6F sheath, by modified Seldinger’s technique, either by a central or by a posterior approach using the anatomical landmarks as a guide. Following sheath placement, St Jude Medical PACEL 6F bipolar pacing catheter (non-balloon tipped rigid electrode) was attached to the pulse generator and introduced through the sheath. Before introducing the lead, a small curve of 70° was made at its tip. The catheter was advanced till paced rhythm was recorded on surface ECG. Subsequently, a pacing threshold was found and if it was below 2 mA, the lead was fixed with the neck. The protocol was made to cannulate right IJV initially; if two attempts were unsuccessful, then left IJV was attempted and if left IJV could not be cannulated in two attempts, then the subclavian vein was punctured. However, the final decision was left to the operator to use his best judgment according to the patient’s anatomy and condition. Every patient received an intravenous antibiotic (most commonly ceftriaxone) after the procedure that was continued till temporary pacemaker lead was in situ.

### Data collection and follow-up

At the time of presentation after a brief history and examination, an ECG was done and a decision for temporary pacing was made. After stabilizing the patient, comprehensive history and physical examination was done. Hemogram and chest X-ray were done before shifting the patient to the ward or ICU.

The number of attempts taken for a successful venous puncture, procedural time (time taken from needle puncture till successful pacing), and pacing threshold was recorded. Procedural complications such as local site hematoma or infection, pneumothorax, pericardial effusion, and mortality were assessed. Finally, the position of the pacing lead was assessed after the procedure on X-ray or fluoroscopy.

### Statistics

Continuous variables are expressed as means ± standard deviations. Categorical variables are expressed as frequencies and percentages.

## Results

### Patients

Of 1093 patients with a mean age of 63.14 ± 9.54 years, 687 (62.85%) were male and 406 (37.15%) were female. The underlying cause was myocardial infarction in 503 (46.02%), degenerative block/bradycardia in 448 (40.98%), drug-induced bradycardia in 118 (10.80%), and End of Life (EOL)/permanent pacemaker failure in 24 (2.20%) patients (Table 1).

**Table 1:** Underlying cause for temporary pacing (*N* = 1093)

Underlying cause	Number of patients (*n*; %)
Myocardial infarction	503 (46.02%)
Degenerative block/bradycardia	448 (40.98%)
Drug-induced bradycardia	118 (10.80%)
EOL/permanent pacemaker failure	24 (2.20%)

### Primary endpoint

The procedure was successful in all but one patient who had stenosis of the brachiocephalic vein due to previous pacemaker leads.

### Secondary endpoint

Right internal jugular access was achieved in 981 (89.75%) patients. Left internal Jugular access was required in 83 (7.59%) patients, whereas subclavian vein access was required in 28 (2.56%) patients. In IJV access, central approach was used in 973 (91.45%) patients and posterior approach was used in 91 (8.55%). In 9 patients, subclavian vein cannulation was directly attempted rather than first using IJV and in 7 patients subclavian vein was cannulated immediately after 1 unsuccessful attempt in IJV cannulation because of short neck and obesity. The mean procedural time was 11.5 ± 2.1 min, and the mean number of attempts taken for achieving successful central venous accesses was 1.54 ± 0.85; however, in the majority central venous access was achieved, 726 (66.42%) patients, in the first attempt (Table 2).

**Table 2:** Approach used and number of attempts taken till successful puncture (*N* = 1093)

No. of attempts	Right IJV	Left IJV	Subclavian vein
1	717 (65.59%)		9 (0.82%)
2	174 (15.91%)		7 (0.64%)
3	90 (8.23%)	65 (5.95%)	
4		18 (1.65%	6 (0.55%)
5			6 (0.55%)
Total	981 (89.75%)	83 (7.59%)	28 (2.56%)

Position of temporary pacing lead was identified on X-ray chest or on fluoroscopy done at the time of permanent pacemaker implantation. Pacing lead tip was located in the right ventricular (RV) cavity abutting interventricular septum or free wall in 843 (77.20%), RV apex in 207 (18.96%) patients, and in right ventricular outflow tract (RVOT) in 42 (3.85%) (Table 3).

**Table 3:** Site of pacemaker lead tip after achieving successful pacing (*N* = 1092)

Site of temporary pacemaker lead tip	Number of patients (*n*; %)
Abutting interventricular septum/RV free wall	843 (77.20%)
Right ventricular apex	207 (18.96%)
RVOT	42 (3.85%)

### Safety outcomes

Overall, 117 (10.70%) patients developed complications (Table 4); however, most of them were minor. Local hematoma developed in 37 (3.38%) patients that were managed conservatively without any further complication. An arterial puncture, while attempting IJV/subclavian vein cannulation, occurred in 25 (2.29%) patients. Pneumothoraxes developed in 12 (1.1%) patients; however, only 2 (0.18%) patients required Intercostal tube insertion; the rest were managed conservatively. Subcutaneous emphysema occurred in 5 (0.46%) patients. Puncture site infection developed in 5 (0.46%) patients, and sepsis or systemic infections developed in 2 (0.18%) patients; all patients were managed successfully with intravenous antibiotics. Ventricular tachycardia (VT) occurred during insertion in 9 (0.82%) patients; all were non-sustained VT and resolved of their own. Pericardial effusion was seen in 21 (1.92%) patients; most of the time, it was mild (< 10 mm) and only 2 patients had moderate effusion (10–20 mm).

**Table 4:** Complications (*N* = 1093)

Complications	Number of patients (*n*; %)
Local hematoma	37 (3.38%)
Arterial puncture	25 (2.29%)
Pneumothorax	12 (1.1%)
Subcutaneous emphysema	5 (0.46%)
Vascular perforation due to lead or sheath	1 (0.09%)
Puncture site infection	5 (0.46%)
Sepsis or systemic infection	2 (0.18%)
VT during insertion	9 (0.82%)
Pericardial effusion	21 (1.92%)
Total	117 (10.70%)

Vascular perforation due to lead or sheath developed in 1 (0.09%) patient. Right IJV perforation occurred due to temporary pacing lead insertion in a patient who had permanent pacemaker implantation done 9 years back and had developed right subclavian and brachiocephalic vein stenosis. In this patient, temporary pacing could not be achieved by jugular or subclavian vein, so a femoral vein approach was required. No mortality attributable to the procedure happened.

## Discussion

As the number of cardiologists and imaging facilities at primary or secondary healthcare facilities is inadequate to cater to the majority of the population, crucial time is lost in transferring patients in need of temporary pacing to tertiary care centers, which leads to increased mortality. This can be prevented if temporary pacing can be done without any imaging guidance by clinicians working in those facilities. We sought to evaluate the safety, efficacy, and practicability of blind temporary pacemaker insertion by cardiology fellows.

Usually, internal jugular, subclavian, femoral, or brachial veins are used for placement of temporary pacemaker lead and access site may depend on physician preference and experience. The right IJV and the left subclavian veins are often preferred, because of the highest rates of proper placement in code situations [3, 4]. Our study found that the cardiology fellows in training can perform this procedure without any imaging guidance with great success and a low complication rate. In our study, the order of preference for central cannulation was right IJV followed by left IJV and then subclavian vein because of easy access in IJV. In total, 982 (89.75%) patients could be cannulated via the right IJV vein and the majority of them, 717 (65.59%), were successful in the first attempt. In some patients, subclavian vein access was preferred because of short necks or obesity.

Only in one patient, temporary pacing could not be achieved by a blind approach. Even though the IJV cannulation was successfully achieved in the second attempt, the pacing lead did not go through easily. On venogram, subclavian and brachiocephalic stenosis was found and femoral access was required. Zhong C et al. in their study failed to pace one among 95 patients in the unguided group, which was later found to have Ebstein anomaly. In the rest 94 patients, successful pacing was achieved in 97% in the first attempt, and in 3% pacing was achieved on the second attempt [2].

The mean number of attempts taken for achieving venous accesses was 1.54 ± 0.85; however, in the majority of patients, 726 (66.42%), central vein cannulation was successful in the first attempt. Karimi-Sari H et al. in their study found that the mean number of attempts for blinded central venous catheterization group was 1.58 ± 0.64 though it was significantly lower in the ultrasound-guided group (1.12 ± 0.3, *P* < 0.001) [5].

**Fig. 1:**
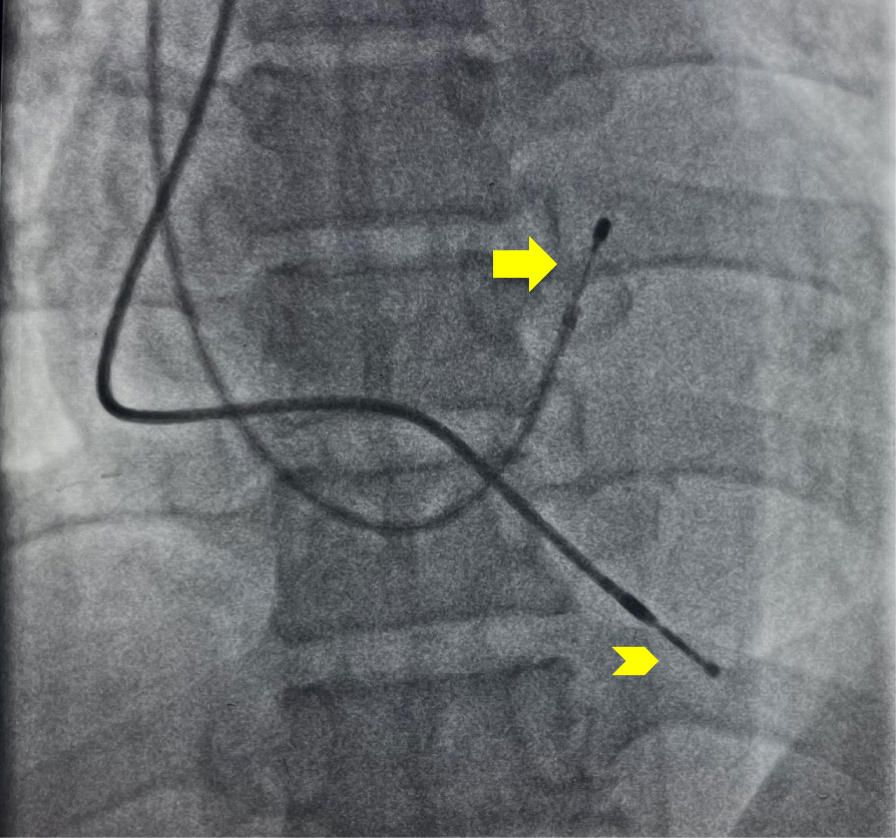
Temporary pacing lead tip (arrow) abutting Interventricular septum and permanent pacing lead (arrowhead at right ventricular apex

Pacing lead tip was located in the RV cavity abutting interventricular septum or free wall in 843 (77.20%) patients. This could be due to the curvature and stiffness of temporary pacing leads. Only in 42 (3.85%) patients, lead tip was found in an unfavorable position of RVOT; however, no sustained arrhythmias occurred in those patients (Fig. 1).

**Fig. 2:**
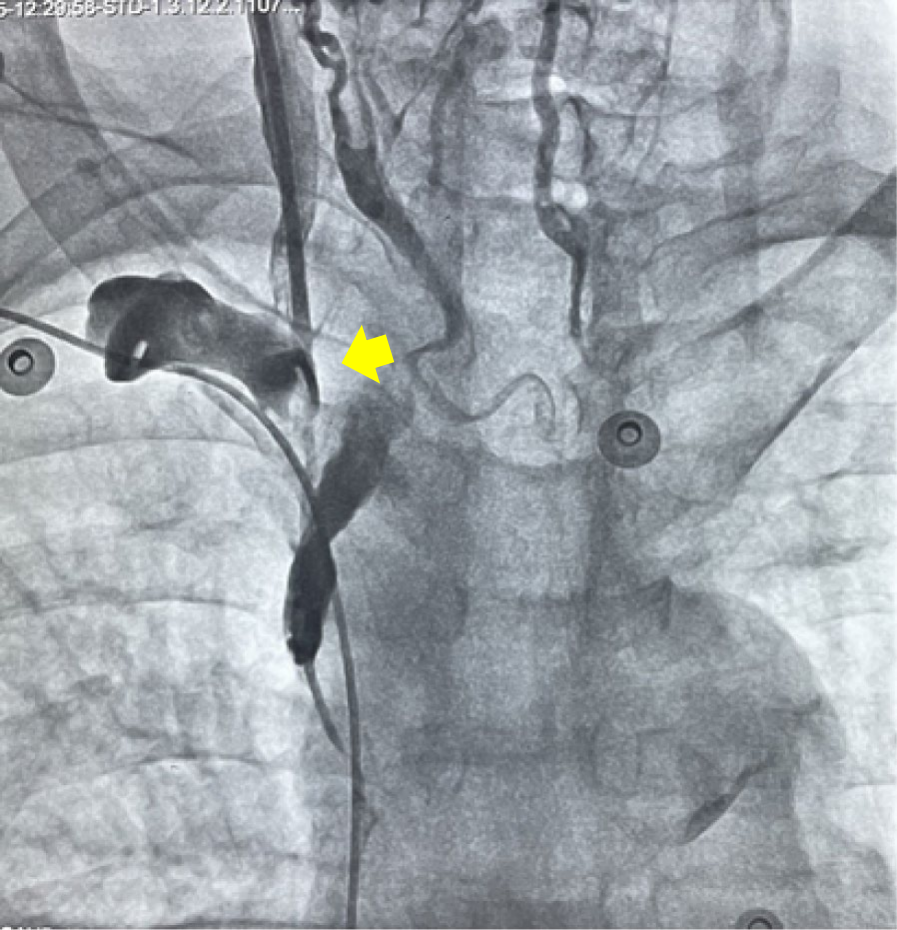
Perforation of right IJV due to temporary pacing lead in a patient with stenosis of right subclavian and brachiocephalic vein after permanent pacing

Though complications developed in 117 (10.70%) patients, most of them were minor. Pneumothoraxes developed in 12 (1.1%) patients; however, only 2 (0.18%) patients required intercostal tube insertion; the rest were managed conservatively. Both these patients were obese and had COPD, and multiple attempts were taken for cannulation. The intercostal tube was removed in one patient on the third day and in another patient on the fifth day. Vascular perforation due to lead developed in 1 (0.09%) patient. Right IJV perforation occurred due to temporary pacing lead in a patient who had developed right subclavian and brachiocephalic vein stenosis after permanent pacemaker implantation was done 9 years back (Fig. 2). In this patient, temporary pacing could not be achieved by jugular or subclavian vein, so a femoral vein approach was required. Subclavian and brachiocephalic vein stenosis has been reported after indwelling devices such as central venous catheters, pacemaker or defibrillator leads, and hemodialysis catheters [6–8]. Venous obstruction has been attributed to thrombus formation or fibrosis caused due to chronic irritation of the endothelium [9]. After device implantation, some amount of venous stenosis has been found in 30–50% of patients [10, 11]. These obstructions are mostly asymptomatic and are usually identified when repeat interventions are needed either to upgrade a device to a dual-chamber or lead replacement. Balloon venoplasty and stenting have been used in selected situations to manage such patients. One must be aware of this complication and be prepared to use a contralateral site for intervention if possible.

Pericardial effusion was seen on echocardiography in 21 (1.92%) patients; most of the time it was mild (10 mm) and only 2 patients had moderate effusion (10–20 mm). It could not be ascertained whether this effusion was because of the procedure or any other cause. However, AK Pradhan et al. found that incidence of pericardial effusion in patients after temporary pacing was 10.0%, but in the majority, 4.2% of cases, pericardial effusion was detected after lead removal, and duration of > 7 days was the only significant predictor of pericardial effusion after temporary pacemaker implantation [12]. No mortality attributable to the procedure happened.

## Conclusions

Even though it is very well known that performing image-guided procedures leads to more successful results and fewer complications, unguided temporary pacing in an emergency setting is possible with high success and a low complication rate. So clinicians working in primary or secondary healthcare facilities and residents should be trained to perform this life-saving procedure at healthcare facilities where image guidance is not available.

However, one needs to be cautious in patients who have underlying pacing leads in place or are known to have congenital abnormalities. In those scenarios, it is preferable to use imaging to prevent any serious complication.

## Data Availability

The dataset used during the current study is available from the corresponding author on reasonable request.

## Abbreviations

IJV: Internal Jugular vein
RVOT: Right ventricular outflow tract
VT: Ventricular tachycardia
RV: Right ventricle
